# Treatment of Maxillary Impacted Canine using Ballista Spring and Orthodontic Wire Traction

**DOI:** 10.5005/jp-journals-10005-1457

**Published:** 2017-02-27

**Authors:** Pradeep Raghav, Kanika Singh, C Munish Reddy, Divya Joshi, Shalu Jain

**Affiliations:** 1 Professor and Head, Department of Orthodontics, Subharti Dental College, Meerut Uttar Pradesh, India; 2 Postgraduate Student (Final Year), Department of Orthodontics, Subharti Dental College, Meerut Uttar Pradesh, India; 3 Professor, Department of Orthodontics, Subharti Dental College, Meerut Uttar Pradesh, India; 4 Postgraduate Student (Final Year), Department of Orthodontics, Subharti Dental College, Meerut Uttar Pradesh, India; 5 Reader, Department of Orthodontics, Subharti Dental College, Meerut Uttar Pradesh, India

**Keywords:** Ballista spring, Canine impaction, Surgical exposure.

## Abstract

In orthodontic practice, impaction of maxillary permanent canines is a frequently encountered clinical problem. After the third molar, the canine is the most frequently impacted tooth. For functional occlusion, bringing the impacted canine into occlusion is important to achieve final esthetic in orthodontic practice. This article illustrates a distinct case in which the three permanent canines maintained their unerupted status till the age of 18 years. The three impacted canines were surgically exposed, attachment bonded, traction given with ballista spring for vertical and labial movement, and ideally positioned with fixed orthodontic mechanotherapy.

**How to cite this article:**

Raghav P, Singh K, Reddy CM, Joshi D, Jain S. Treatment of Maxillary Impacted Canine using Ballista Spring and Orthodontic Wire Traction. Int J Clin Pediatr Dent 2017;10(3):313-317.

## BACKGROUND

The reported incidence of impacted teeth is 0.92^1^ and *1.7%}* According to Bishara,^2^ the most common causes for canine impactions are usually localized and are the result of any one, or a combination of the following factors: (a) Tooth size-arch length discrepancies, (b) prolonged retention or early loss of the deciduous canine, (c) abnormal position of the tooth bud, (d) the presence of an alveolar cleft, (e) ankylosis, (f) cystic or neoplastic formation, (g) dilaceration of the root, (h) iatrogenic origin, and (i) idiopathic condition with no apparent cause. Two most commonly used methods for exposing impacted canine are (1) surgical exposure, allowing natural eruption, and (2) surgical exposure with placement of an auxiliary attachment.^2^ Orthodontic forces are subsequently applied to the attachment to move the impacted tooth. This is a case report that illustrates the effects of Ballista spring for the eruption of palatally impacted canine.

## CASE REPORT

**Figs 1A to H:**
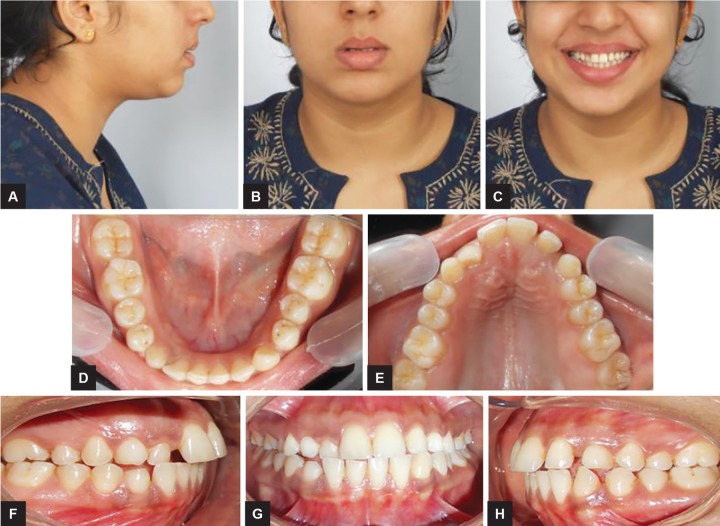
Extra- and intraoral views of 18-year-old female patient with palatally impacted maxillary canines and retained deciduous canines

**Figs 2A to C:**
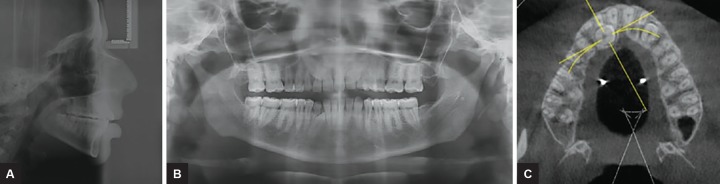
Lateral cephalogram, orthopantomogram, and CBCT view with impacted maxillary canines bilaterally

A 18-year-old female reported with a chief complaint of retained milk teeth in upper and lower front region. On extraoral examination, the profile of patient was convex with normal interlabial gap (Fig. 1). On intraoral examination, she presented with end-on molar relationship on right and left side, with unerupted maxillary canines and mandibular right canine, retained deciduous canine except for left mandibular canine. Palatal bulge was identified in the maxillary left and right palatal region suggestive of the position of the impacted canines. Spacing was present between lateral incisor and canine on left side. Anterior cross-bite and rotation was present with respect to the right lateral incisor with normal overjet and overbite (Fig. 1). Orthodontic records were taken, which included maxillary and mandibular impressions, extraoral and intraoral photographs, lateral cephalogram, orthopantomogram, and cone beam computed tomography (CBCT; Fig. 2). The panoramic radiograph showed all permanent teeth including developing third molar buds and impacted maxillary right and left canine. The left and right maxillary canine were mesially inclined toward the midline with angulation to the midline of 31° and 30° respectively. They were overlapping, the mesial third of the maxillary lateral incisor and as par the sector classification^3^ were in sector IV (Fig 2). The CBCT evaluation of impacted canines was done in relation to adjacent teeth.

**Figs 3A to C:**
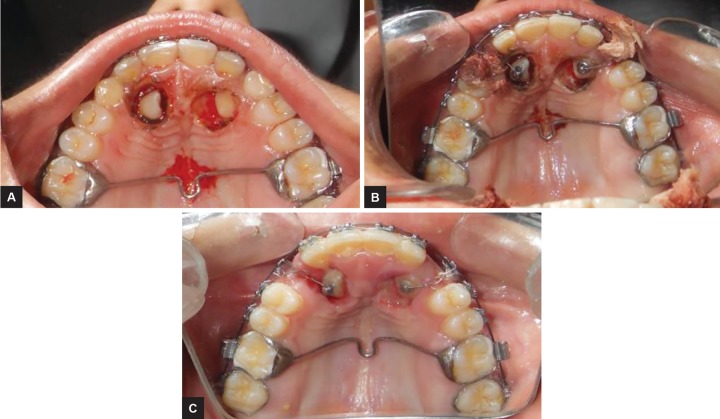
(A) Surgical exposure of maxillary canine using open window technique; (B) lingual button bonded onto the exposed canines; and (C) after 1 week of healing

**Figs 4A to D:**
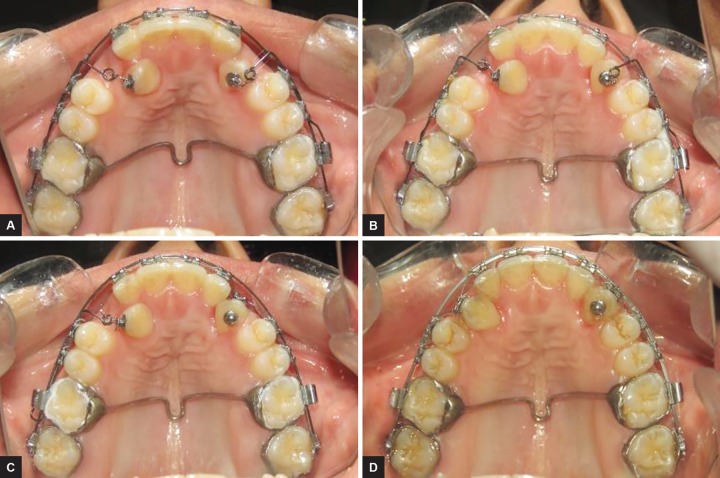
(A) Ballista spring attached after 10 days of healing; (B) after 1 month; (C) after 2 months; and (D) after 3 months

A fixed mechanotherapy with nonextraction treatment was planned for the case. For anchorage preparation, transpalatal arch was soldered to first maxillary molars. The vertical arm of ballista spring with 0.014" Australian wire was attached to the impacted canine to direct a palatal-occlusal force from the buccal side and horizontal arm was ligated into the slot of premolar brackets. In a period of 3 months vertical and labial traction of maxillary canine was achieved using ballista spring (Figs 3 and 4). After 12 months by the dual traction (vertical and labial) of ballista spring, both the canines were fully erupted and almost close to the arch.

## DISCUSSION

**Figs 5A and B:**
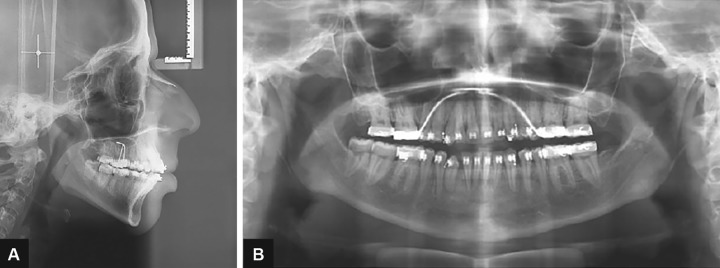
Midstage lateral cephalogram and orthopantomogram showing maxillary impacted canine into the arch

**Figs 6A to C:**
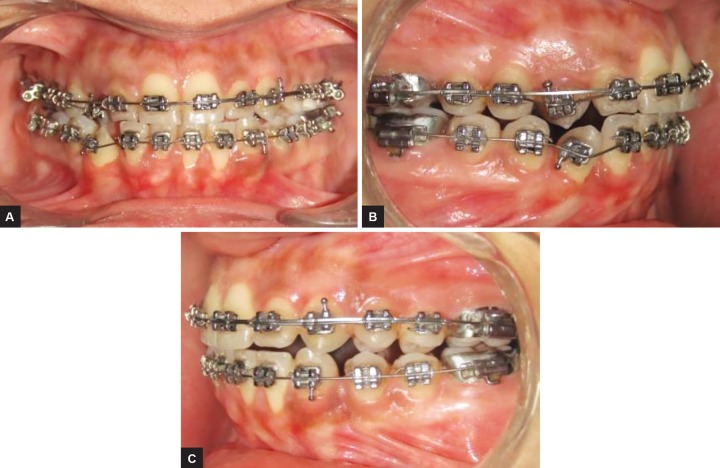
Midstage intraoral photographs showing alignment of canines into the arch

Maxillary canines are the cornerstone of the dental arch and play a very important role in smile esthetics and are essential for maintaining a functional occlusion. Extraction of impacted canine should be avoided. In the present case, metal brackets (American orthodontics) of 0.022" slot were used. After leveling and alignment phase was done by following wire sequence (0.014", 0.016", 0.018" nickel-titanium). This was followed by Stabilizing the maxillary arch with 0.018" Australian wire. Once canines were closer to the main arch, Twin arch wires was used , a 019*025 stainless steel base arch was placed for stabilization along with the 016 NITI wire overlay for bringing the canines into main arch (Figs 5 and 6). A cuspid circle was constructed midway between the lateral incisor and first premolar. Retained deciduous canines were not extracted for maintenance of space between lateral incisor and first premolar. Followed by exposure of the impacted maxillary canine, anesthesia was obtained using block and infiltration injection of 2% lidocaine with 1:100,000 epinephrine. Deciduous canines were extracted in the same appointment with the same protocol before the exposure of canine (Figs 3A to C). As Graber and Vanarsdall^4^ state, as the palate is all masticatory mucosa, graft is not placed on the tooth. So, in this report, the successful exposure of a palatally impacted maxillary canine was performed using the open window technique with electrocautery. This method controls localized bleeding by cauterizing vessels and coagulating blood and provides good visualization of the surgical field.^5^ The advantage of this technique includes vertical traction on the impacted tooth toward the middle of the palate, easy fabrication, less traumatic in comparison to other techniques, and easy to insert and remove.^6^ Then a bonded attachment was placed and tooth movement was initiated.^4^ Ligature wire was attached from the lingual button to the cuspid circle of the main archwire (Figs 3A to C), although in recent years various techniques have been developed for bringing the impacted canine in occlusion.^7^ But, after 1 week of healing, ballista spring was placed. Ballista spring was given by Jacoby^6^ because it has an added advantage over other methods that it could be used before and during leveling and alignment phase. As the patient wanted the treatment to get finished early we used ballista spring. For construction of ballista spring 0.014" round Australian wire was used. Horizontal arm was placed in the slot of premo-lar and molar headgear tube. It stores its energy by being twisted on its long axis. Transpalatal arch was soldered to maxillary first molars to maintain anchorage. To avoid any rotation of the wire in the headgear tube, bends were given just flushing with the headgear tube on the distal and the mesial side. The horizontal arm accumulates the energy when ballista spring is activated and ligated on first and second premolar bracket. It allows to rotate the wire in slot as hinge axis. For vertical arm a 90° bend from middle of the extraction space of deciduous canine was given. The length of the vertical arm is kept 2 mm short from maxillary permanent canine to direct an occlusal force palatally and horizontally.^5^ Hence, we were able to bring the impacted canine into occlusion successfully using ballista spring.

## CONCLUSION

This approach has been successfully used in the current case in terms of adequate attached gingival tissue preservation around the disimpacted canine. Hence, it could be stated that within 3 to 4 months, ballista spring can show a great success for treating impacted maxillary canine.
